# Virtual and Augmented Reality for Cognitive and Language Rehabilitation in Children and Adolescents: A Scoping Review

**DOI:** 10.3390/healthcare14193299

**Published:** 2026-10-03

**Authors:** Maria Pagano, Irene Cappadona, Anna Anselmo, Alessio Codispoto, Davide Cardile, Rosaria De Luca, Angelo Quartarone, Rocco Salvatore Calabrò, Francesco Corallo

**Affiliations:** IRCCS Centro Neurolesi Bonino-Pulejo, 98124 Messina, Italyalessio.codispoto10@gmail.com (A.C.);

**Keywords:** virtual reality, cognitive rehabilitation, executive functions, attention, language rehabilitation, children, adolescents

## Abstract

**Highlights:**

**What are the main findings?**

- Virtual reality (VR) showed the strongest evidence for attention and executive functions.
- Studies used different levels of immersion, but the available evidence does not permit conclusions regarding their comparative effectiveness.

**What are the implications of the main findings?**

- VR may be a useful adjunct to pediatric rehabilitation.
- Larger trials with standardized outcomes and follow-up are needed.

**Abstract:**

**Background:** Virtual reality (VR) is increasingly used in pediatric rehabilitation to deliver engaging, adaptive, and ecologically relevant interventions. **Objective:** To map the available evidence on VR interventions targeting cognitive and linguistic functions in pediatric populations, focusing on intervention characteristics, technologies, outcome domains. **Methods:** A scoping review was conducted across PubMed, Web of Science, Cochrane Library, IEEE Xplore, Embase, PsycINFO and Scopus without publication-date restrictions. English-language journal articles involving participants younger than 18 years were considered. Eligible interventions targeted attention, executive functions, memory, processing speed, language, speech, or related cognitive-linguistic abilities. Methodological quality was assessed using design-specific Joanna Briggs Institute critical appraisal checklists. **Results:** Twelve studies were included. Considerable heterogeneity was observed in populations, treatment intensity, comparator conditions, technologies, and outcome measures. The most consistent benefits concerned attention and executive functions, particularly inhibitory control, cognitive flexibility, working memory, planning, processing speed, and metacognition. Evidence for language, memory, and social cognition was promising but more limited. Positive findings were reported across immersive, semi-immersive, and non-immersive VR systems, with no clear evidence of superiority according to level of immersion. Follow-up assessments were uncommon, and methodological quality was heterogeneous across study designs. **Conclusions:** VR interventions appear promising for pediatric cognitive and language rehabilitation. Larger controlled studies using standardized outcomes, active comparators, and longer follow-up are needed.

## 1. Introduction

Virtual reality (VR) is increasingly used in rehabilitation to create interactive, controllable, and ecologically meaningful environments in which patients can practice clinically relevant activities. VR is a computer-based technology that generates artificial two- or three-dimensional environments with which users can interact in real time through visual, auditory, haptic, or motion-based information. In rehabilitation, these environments may support repeated, goal-directed practice, immediate feedback, task adaptation, and active engagement [1,2,3]. Depending on the technological configuration, the virtual environment may partially or almost completely replace sensory information from the physical world [3]. Augmented reality (AR) represents a related but distinct technology in which digital information or virtual objects are superimposed on the real environment rather than replacing it entirely [4]. Thus, whereas VR primarily immerses the user in a simulated environment, AR preserves direct visual contact with the physical world while enriching it with digital elements. Although both technologies may be applied in rehabilitation, VR currently represents the more extensively studied approach in cognitive and neuropsychological rehabilitation [5,6].

Within this framework, cognitive rehabilitation can be defined as a structured set of therapeutic interventions designed to improve cognitive functioning through restorative and compensatory strategies [7]. Restorative approaches aim to strengthen or re-establish impaired cognitive processes through repeated and progressively challenging practice, whereas compensatory approaches seek to improve everyday functioning by introducing strategies, environmental modifications, external aids, or alternative ways of performing a task [8]. Cognitive rehabilitation therefore extends beyond memory or attention training and may involve attention, learning and memory, executive functions, reasoning and problem solving, processing speed, visuospatial and perceptual abilities, awareness, language, speech-related cognitive processes, and functional communication [9,10]. Language and communication are particularly relevant within this framework because receptive and expressive language, lexical retrieval, verbal learning, morphosyntactic processing, discourse, and pragmatic communication rely on the interaction of linguistic and broader cognitive processes and can substantially influence academic performance, social participation, and autonomy [11].

The term VR-based rehabilitation broadly refers to therapeutic assessment or training delivered through interaction with computer-generated environments [12]. Compared with conventional paper-and-pencil or therapist-led exercises, VR can reproduce functional situations that would be difficult to standardize, repeat, or safely recreate in routine clinical settings [13]. Virtual environments also permit automatic recording of performance and real-time modification of task characteristics according to the patient’s responses. Immediate multimodal feedback, graded difficulty, repetition, reward mechanisms, and individualized progression may promote learning and engagement while reducing the monotony often associated with intensive rehabilitation [5,10]. These features are especially relevant in cognitive rehabilitation, in which treatment commonly requires sustained attention, repeated practice, progressive adaptation, and opportunities to apply acquired strategies to increasingly complex situations [13,14].

VR systems are commonly classified according to their degree of immersion. Immersion refers to the extent to which the system redirects the user’s sensory experience from the physical to the virtual environment [15]. Non-immersive VR includes screen-based systems, such as desktop computers, tablets, or smartphones, in which users remain aware of the surrounding physical environment while interacting with virtual content or avatars. Semi-immersive VR uses larger projection or motion-tracking systems, whereas immersive VR substantially replaces the user’s visual field, typically through head-mounted displays or surrounding projection environments [5,16,17,18,19]. In this context, a system does not need to fully isolate the user from the physical world to qualify as VR; screen- or smartphone-based interaction with a computer-generated virtual environment or avatar may therefore be classified as non-immersive VR [17]. AR can be considered complementary in this context because it allows rehabilitation tasks to integrate virtual stimuli with real-world objects and spaces rather than replacing the surrounding environment [20].

The rationale for using VR in cognitive rehabilitation extends beyond technological novelty. Effective rehabilitation generally requires meaningful, repetitive, progressively challenging, and goal-directed practice adapted to the individual’s residual abilities and functional needs. Virtual environments can incorporate these principles into simulations of classrooms, homes, streets, shops, social encounters, or game-like scenarios [21]. This makes it possible to train attention, inhibitory control, cognitive flexibility, planning, working memory, episodic and prospective memory, processing speed, visuospatial abilities, and other executive or cognitive processes within dynamic tasks rather than isolated exercises [22,23]. The ability to manipulate environmental complexity and provide immediate feedback may also facilitate the progressive approximation of real-world cognitive demands. Nevertheless, the ecological validity of a virtual task does not necessarily guarantee transfer to everyday functioning, and the generalization of trained abilities remains an important issue in rehabilitation research [24].

The same principles are applicable to language, speech, and cognitive-communication rehabilitation. Virtual environments can provide structured visual and auditory stimuli for receptive-language activities, naming and lexical retrieval, repetition, phonological processing, morphosyntactic training, sentence formulation, narrative production, and functional communication [25]. They can also recreate communicative situations in which users practice conversation, turn-taking, interpretation of social cues, emotion recognition, perspective-taking, and pragmatic language. VR therefore offers a potential framework for integrating cognitive and linguistic processes within the same rehabilitation environment, an aspect that may be particularly relevant during development because language functions are closely interconnected with attention, working memory, executive control, and learning [26]. Reviews of VR in communication disability have highlighted the potential of virtual environments to provide structured and repeatable communication opportunities, although the field remains methodologically heterogeneous [27].

The pediatric population may particularly benefit from VR-supported rehabilitation. Children and adolescents often require intensive and repetitive training but may experience fatigue, frustration, attentional fluctuations, or reduced motivation during conventional treatment. Game-like tasks, immediate feedback, progressive goals, multisensory stimulation, and interactive scenarios may improve engagement and adherence and allow repetitive exercises to be presented as meaningful challenges [28]. VR may also offer the opportunity to practice cognitive or communicative abilities in simulated contexts while limiting the real-world consequences of errors. Portable and remotely deliverable systems may further support continuity of care and extend rehabilitation beyond specialized clinical settings, although these applications require appropriate professional supervision, caregiver involvement, reliable technology, and careful monitoring of treatment fidelity [29].

At the same time, the use of VR in children and adolescents requires specific attention to physical tolerability and safety because visual, vestibular, sensory, motor, and cognitive systems are still developing [30]. The most frequently described adverse effect associated with immersive VR is cybersickness, which may include nausea, dizziness, disorientation, headache, sweating, fatigue, visual discomfort, and sensations resembling motion sickness [31]. These symptoms are thought to result, at least in part, from sensory conflict between visually perceived movement and vestibular or proprioceptive information [32]. Head-mounted systems may also produce eye strain, head or neck discomfort, and transient changes in postural stability [33]. Such effects may be particularly relevant in younger children, who may have greater difficulty recognizing, describing, or spontaneously reporting emerging symptoms.

Available evidence generally suggests that short and supervised VR exposure is well tolerated in children, but knowledge regarding prolonged, repeated, or intensive exposure remains limited [34]. Pediatric safety research has highlighted the need to monitor visual discomfort, headache, dizziness, nausea, fatigue, head and neck discomfort, and balance changes during and after VR use [35]. A systematic review of head-mounted VR in children also found that adverse effects were predominantly mild, while emphasizing inconsistent safety reporting, limited use of validated pediatric symptom measures, and insufficient evidence regarding repeated exposure [34]. Consequently, appropriate session duration, scheduled breaks, age-appropriate equipment, careful adjustment of headset fit, screening for visual or vestibular vulnerabilities, and systematic monitoring of adverse symptoms should be considered when VR is implemented in pediatric rehabilitation. Evidence concerning comparable physical adverse effects of AR in pediatric rehabilitation is currently more limited.

Despite increasing interest, the available literature remains fragmented across clinical populations, cognitive and linguistic domains, technological platforms, levels of immersion, treatment intensity, and outcome measures. Cognitive and language rehabilitation have also frequently been investigated as separate therapeutic areas, despite the close interaction between attention, executive functions, memory, learning, and language during development. In addition, differences in study design, intervention dose, comparator conditions, assessment instruments, and follow-up limit straightforward comparison across studies.

Accordingly, this scoping review aims to map and critically characterize VR-based cognitive and linguistic rehabilitation in children and adolescents, examining intervention characteristics, technologies, outcome domains, and methodological quality, while considering AR as a related technology.

## 2. Materials and Methods

### 2.1. Search Strategy and Inclusion Criteria

This scoping review was designed according to the methodological framework for scoping reviews proposed by the Joanna Briggs Institute (JBI) [36] and reported in accordance with the Preferred Reporting Items for Systematic Reviews and Meta-Analyses extension for Scoping Reviews (PRISMA-ScR) [37] and related checklist is available in the Supplementary Material (Table S5). The protocol for this scoping review was registered on the Open Science Framework (OSF) and is available at: https://doi.org/10.17605/OSF.IO/JVZBU. The review question and eligibility criteria were structured according to the Population–Concept–Context (PCC) framework. The population of interest comprised children and adolescents younger than 18 years; the concept concerned virtual reality (VR)-based cognitive and linguistic rehabilitation, with augmented reality (AR) also considered when used as a related rehabilitation technology; and the context included clinical, rehabilitation, neurodevelopmental, neurological, psychiatric, and educational-health settings.

A comprehensive literature search was conducted in PubMed/MEDLINE, Web of Science Core Collection, Cochrane Library, IEEE Xplore, Embase, PsycINFO and Scopus. No restriction on publication date was applied. Searches were limited to journal articles published in English. Search strategies were adapted to the syntax and controlled vocabulary of each database and combined terms referring to three main conceptual blocks: (1) the pediatric population; (2) cognitive, linguistic, and cognitive-communication rehabilitation; and (3) VR or AR technologies.

The pediatric search block included terms such as child, adolescent, pediatric, paediatric, youth, and related variants. The rehabilitation block included general terms such as cognitive rehabilitation, cognitive remediation, cognitive training, neuropsychological rehabilitation, language rehabilitation, speech therapy, and communication training, together with specific cognitive and linguistic domains combined with rehabilitation-related terms. The technology block included terms related to virtual reality, virtual environments, immersive, semi-immersive and non-immersive virtual reality, head-mounted displays, motion-based virtual systems, augmented reality, and related technologies. Boolean operators and proximity operators were used where supported by the individual database. The complete database-specific search strategies are reported in the Supplementary Material (Table S1).

Studies were considered eligible when they: (1) included children or adolescents younger than 18 years, or provided separately extractable data for participants within this age range; (2) investigated an intervention based primarily on VR, or on AR as a related technology; (3) used a clearly identifiable VR/AR technology allowing interaction with a computer-generated or digitally augmented environment; (4) were conducted within an explicitly defined clinical or rehabilitation context; (5) presented original empirical data and had cognitive or speech/language rehabilitation as an explicit intervention objective; (6) reported outcomes relevant to the targeted cognitive or speech/language domain; and (7) were published as full-text journal articles in English.

Studies were excluded if they involved exclusively participants aged 18 years or older; used VR/AR solely for assessment without a therapeutic or training component; targeted exclusively motor, balance, physical, or sensory rehabilitation without a relevant cognitive or linguistic outcome; did not include a clearly identifiable VR/AR technology; were conducted exclusively in educational contexts without a rehabilitation purpose, or lacked an explicit cognitive, linguistic, or cognitive-communication rehabilitation objective; or used conventional videogames or digital interventions without a clearly identifiable VR or AR component. In addition, reviews, meta-analyses, protocols, editorials, letters, conference abstracts, conference proceedings, book chapters, dissertations, or other non-journal publications were excluded.

### 2.2. Study Selection and Quality Assessment

All records retrieved from the electronic databases were imported into Microsoft Excel 2021 (Microsoft Corporation, Redmond, WA, USA). The final database search was conducted on 12 June 2026. Study selection was performed in two sequential stages. First, titles and abstracts were screened against the predefined eligibility criteria. Articles considered potentially relevant, or for which eligibility could not be determined from the title and abstract alone, were retrieved for full-text evaluation. Second, the full texts were independently assessed against the inclusion and exclusion criteria. Two reviewers (M.P. and I.C.) independently performed the screening process, and disagreements were resolved through discussion; when consensus could not be reached, a third reviewer was consulted. The overall study-selection process was summarized using a PRISMA flow diagram, reporting the number of records identified, duplicates removed, records screened, full texts assessed, and studies finally included (Figure 1). Data were independently extracted by two reviewers (M.P. and I.C.) using a standardized data-charting framework and included study population, design, rehabilitation setting, VR/AR technology and level of immersion, intervention characteristics, rehabilitation target, outcome measures, main findings, and limitations. Disagreements were resolved by discussion and, when necessary, consultation with a third reviewer (A.A.).

Methodological quality was evaluated to support interpretation of the available evidence and to distinguish findings derived from stronger experimental designs from those obtained in exploratory or uncontrolled studies. JBI design-specific critical appraisal checklists were used according to the methodological characteristics of each study. Quality assessment was performed independently by two reviewers (M.P. and I.C.). Each JBI item was rated as “Yes,” “No,” “Unclear,” or “Not applicable.” Disagreements were resolved by discussion and, when necessary, consultation with a third reviewer (A.A.).

For studies involving authors who were also members of the review team, data extraction and methodological appraisal were independently verified by reviewers who were not authors of the corresponding publication.

## 3. Results

A total of 12 studies met the inclusion criteria and were included in the final synthesis. Table 1 summarizes the characteristics and findings of the included studies.

### 3.1. Overall Results

The included studies were published between 2002 and 2026 and were conducted across Europe, Asia, Australia, and the United States. Sample sizes ranged from single-case reports to randomized controlled trials involving up to 137 participants overall in Lyu et al. [46]. The populations examined were clinically heterogeneous and included children and adolescents with attention-deficit/hyperactivity disorder (ADHD), autism spectrum disorder (ASD), traumatic brain injury (TBI), developmental language disorder, specific learning disorders, depressive episodes, and children with developmental coordination disorder receiving AR- or VR-based executive function training. Cappadona et al. [43] and De Domenico et al. [49] were treated as separate samples for the purposes of the present review. Both studies were conducted within the same clinical rehabilitation context, with partially overlapping recruitment periods. However, because individual participant identifiers were not available in the anonymously reported data, complete participant-level independence could not be independently verified. Study designs included randomized controlled trials, pilot randomized trials, uncontrolled pre-post studies, feasibility studies, and single-case designs. Across studies, the aims focused on improving attention, executive functions, memory, language, social cognition, or broader cognitive functioning through VR- or, less frequently, AR-based rehabilitation. The interventions varied considerably in duration and intensity, ranging from short programs delivered over two weeks to interventions extending across six months. Standardized outcome measures included neuropsychological tests of attention, executive functioning, working memory, processing speed, and social cognition, as well as standardized language assessments and parent-reported measures of everyday executive functioning. Overall, most studies reported improvement in at least one targeted cognitive or linguistic domain, although findings were less consistent for generalized or everyday functional outcomes [38,39,40,41,42,43,44,45,46,47,48,49].

### 3.2. Quality Assessment Results

Methodological quality was heterogeneous across study designs. Randomized controlled trials showed strengths in the use of standardized outcome measures and statistical analyses, but recurrent limitations included unclear allocation concealment, limited blinding, and the frequent use of inactive control conditions. Uncontrolled and single-case studies showed greater susceptibility to confounding, practice effects, and limited generalizability. Follow-up assessments were uncommon across the evidence base and methodological quality was heterogeneous across study designs. Overall, the quality appraisal supports cautious interpretation of the positive findings and highlights the need for larger, well-controlled trials with clearer methodological reporting and longer-term follow-up. The methodological quality assessment of the included studies is reported in the Supplementary Materials (Tables S2–S4).

### 3.3. Comparison According to Intervention Characteristics

Comparison of the studies according to intervention characteristics revealed substantial heterogeneity in treatment duration, frequency, therapeutic intensity, and intervention content. Findings from uncontrolled studies were interpreted as within-group pre-post changes and not as evidence of comparative efficacy. Evidence of superiority was reserved for controlled between-group comparisons, while feasibility and safety outcomes were reported separately. The included studies investigated VR/AR-based interventions across different neurodevelopmental conditions, including autism spectrum disorder, developmental coordination disorder, speech and language disorders, and cognitive impairments. Interventions targeted different domains, including executive functions, attention, cognitive skills, and language abilities. Cho et al. [38], for example, delivered eight sessions over two weeks, whereas Zhao et al. [45] used a four-week program comprising 12 sessions. Intermediate-duration interventions were more common, including ten sessions over five weeks in the social-cognition program of Didehbani et al. [39] and 20 sessions over seven weeks in the cognitive-training intervention of Lyu et al. [46]. By contrast, interventions targeting language rehabilitation were markedly longer: Cappadona et al. [43] and De Domenico et al. [49] delivered two 60 min sessions per week over six months, corresponding to 48 treatment sessions. Thus, language interventions were characterized by a considerably greater cumulative treatment exposure than most cognitive-training protocols.

Differences were also evident in the specificity of therapeutic content. Some interventions focused predominantly on a circumscribed cognitive process, such as attention [38], whereas others simultaneously addressed several executive components, including inhibition, flexibility, working memory, planning, and processing speed [44,46]. The interventions targeting DLD involved a broader linguistic rehabilitation program addressing receptive and expressive language rather than isolated language tasks [39,43]. Other studies adopted multimodal interventions in which VR/AR was combined with physical exercise, motor interaction, conventional rehabilitation, or social-cognitive activities [41,45]. Consequently, the contribution specifically attributable to the virtual component was easier to interpret in interventions focused primarily on cognitive or language training than in multimodal programs.

Additional interventions included an AR-based cognitive rehabilitation program for children with ASD [47] and a semi-experimental Kinect-based VR training program targeting executive functions in children with developmental coordination disorder [48].

The nature of the comparison condition further differentiated the evidence. Some randomized studies compared VR-supported rehabilitation directly with conventional treatment, providing a more clinically informative estimate of its added value [43,49]. Other trials used no-intervention or waitlist control groups [38,46]. These differences are relevant because improvements relative to an inactive comparator may reflect not only the specific effects of VR but also greater treatment exposure, therapist interaction, physical activity, novelty, or motivation.

Follow-up assessment was comparatively uncommon. Most studies measured outcomes immediately after completion of the intervention, limiting conclusions regarding durability. Di Giusto et al. [44] represented an important exception, with several improvements in executive functioning remaining evident at six months. Overall, comparison across intervention characteristics did not indicate a clear dose–response relationship. Longer treatment was not consistently associated with stronger comparative effects; for example, the prolonged language intervention of Cappadona et al. [43] showed advantages for several linguistic outcomes, whereas the later larger trial by De Domenico et al. [49], despite a comparable treatment schedule, did not demonstrate clear superiority over conventional treatment. The findings therefore suggest that treatment effectiveness is likely influenced by the interaction among intervention content, clinical population, comparator, outcome sensitivity, and treatment intensity rather than by session number alone.

### 3.4. Comparison According to VR/AR Technology and Level of Immersion

Comparison according to technological characteristics showed that potentially beneficial findings were reported in studies using immersive, semi-immersive, and non-immersive systems; however, no included study directly compared levels of immersion under equivalent conditions.

Immersive VR was used in some of the earliest and more neurologically oriented studies. Cho et al. [38] employed a head-mounted display-based environment for attention training and reported greater attentional improvement than non-VR computerized training and no intervention. Similarly, De Luca et al. [41] used an immersive projection-based environment with motion capture in an adolescent with severe TBI and reported improvements across several cognitive and behavioral functions. However, the evidence for immersive VR was limited by the small samples and, in some cases, single-case methodology.

Semi-immersive VR was more frequently integrated with body movement and motion tracking. Di Giusto et al. [44] used a projection- and motion-tracking environment and observed improvements across several executive domains, including attention, inhibition, flexibility, and planning. Thus, semi-immersive systems tended to combine cognitive demands with active sensorimotor interaction, making them particularly suited to cognitive-motor or executive-function training.

Non-immersive systems were more prominent in language, social ability training, and cognitive rehabilitation. Didehbani et al. [39] used a desktop avatar-mediated virtual environment to train social cognition in children with ASD, whereas Cappadona et al. [43] and De Domenico et al. [49] used touchscreen-based virtual rehabilitation for children with DLD. Despite their lower level of sensory immersion, these systems enabled structured, repetitive, and interactive cognitive-linguistic activities. The favorable findings observed in these studies indicate that high immersion is not necessary for a system to support rehabilitation and that non-immersive technology may be particularly appropriate for younger children, in whom accessibility, ease of interaction, tolerability, and therapist supervision may be especially important.

AR was considerably less represented than VR. Zhao et al. [45] used an AR-based cognitive-physical intervention in children with ADHD and reported improvements in metacognition, global executive functioning, and ADHD symptoms. Although promising, a single study does not permit comparison of AR effectiveness with VR.

Taken together, the available studies do not support a hierarchical relationship whereby immersive VR is systematically superior to semi- or non-immersive systems. Rather, technology appeared to be selected according to the therapeutic objective: immersive environments were mainly used for attention and complex neurorehabilitation, motion-based semi-immersive systems for executive and cognitive-motor training, and non-immersive systems for language and social-cognitive interventions. The absence of direct comparisons between different levels of immersion prevents conclusions regarding comparative effectiveness. Table 2 provides an overview of the distribution of VR/AR technologies across the included pediatric populations, together with the main rehabilitation outcomes investigated.

### 3.5. Comparison According to Outcome Domains and Assessment Measures

Comparison according to the targeted domains showed that attention and executive functions constituted the largest and most consistently investigated area. Attention was targeted either directly [38] or as part of broader executive-function interventions. Improvements in attentional control were also observed in children with specific learning disorders, TBI, ADHD, and depressive episodes [41,44,45,46]. Executive-function interventions addressed heterogeneous components including inhibitory control, cognitive flexibility, planning, working memory, processing speed, and metacognition [44,45,46]. Across these studies, improvements were commonly found in at least some executive domains, but benefits were not necessarily generalized across all executive outcomes within the same trial.

A relevant distinction emerged between performance-based neuropsychological outcomes and measures of broader everyday functioning. Studies using tasks directly related to the trained process, such as continuous performance measures, inhibition tasks, card-sorting tests, processing-speed measures, or cognitive batteries, frequently detected post-intervention changes [38,44,46]. Questionnaire-based measures of everyday executive functioning also showed improvements in some studies, particularly Zhao et al. [45], but findings across the literature were less uniform. This pattern suggests that VR-related gains may be more consistently detected as near-transfer effects, involving the trained or closely related cognitive process, than as broader changes in everyday functioning.

Memory was much less frequently examined as a primary domain. Cooper et al. [40] focused specifically on autobiographical and prospective memory in a single adolescent and reported improvements in event recall and perceived everyday memory functioning. Working memory, however, was frequently incorporated within broader executive interventions, particularly in ADHD and depressive populations [45,46]. The evidence base for isolated memory rehabilitation therefore remains considerably smaller than that for executive functions.

Language represented another distinct domain. Cappadona et al. [43] reported improvements across multiple receptive and expressive language components, including comprehension, naming, morphosyntax, sentence construction, and spontaneous language production, with greater gains for several outcomes in children receiving VR-supported treatment. In contrast, De Domenico et al. [49] found improvement over time in both VR-supported and conventional therapy groups but no clear comparative superiority of VR across the assessed language domains. The comparison of these studies suggests that virtual systems are feasible for delivering pediatric language rehabilitation, but the extent to which they enhance effectiveness beyond conventional speech-language therapy remains uncertain.

Finally, social cognition was primarily investigated in children with ASD. Didehbani et al. [39] reported improvements in emotion recognition, social attribution, and analogical reasoning following virtual social-cognition training. Overall, comparison across domains indicates that the evidence is most substantial for attention and executive functioning, whereas memory, language, and social cognition have been investigated in fewer studies. Moreover, the diversity of standardized instruments used across studies limits direct quantitative comparison. The available findings suggest that the clearest intervention effects occur on measures closely aligned with the trained cognitive or linguistic function, while evidence for transfer to broader functional outcomes remains comparatively limited.

## 4. Discussion

This scoping review mapped the available evidence on VR-based cognitive and linguistic rehabilitation in children and adolescents, while also considering AR when used as a related technology. The inclusion of recent studies further expands the emerging evidence on technology-assisted cognitive rehabilitation, including AR-based cognitive training in children with ASD and semi-immersive VR interventions targeting executive functions in children with developmental coordination disorder. The literature was highly heterogeneous with respect to clinical populations, study designs, intervention dose, comparison conditions, technologies, and outcome measures. The most consistent findings concerned attention and executive functions, including inhibitory control, cognitive flexibility, working memory, planning, processing speed, and metacognition, whereas memory, language, social skills and cognitive rehabilitation were investigated less frequently. In addition, potentially beneficial findings were observed across immersive, semi-immersive, and non-immersive VR systems, with no clear evidence from the included studies that a higher level of immersion was associated with superior rehabilitation effects. Benefits were more consistently observed on measures closely related to the trained cognitive or linguistic function than on broader functional outcomes, while long-term maintenance was insufficiently investigated. These findings support VR as a promising adjunctive approach for pediatric cognitive and cognitive-linguistic rehabilitation, but they also indicate that the evidence remains insufficiently standardized to establish which patients, treatment protocols, or technological configurations are most likely to benefit.

Recent studies included in this review further support the potential application of VR/AR technologies in neurodevelopmental populations. Abdullah et al. [47] investigated an AR-based cognitive rehabilitation approach in children with autism spectrum disorder, while Hashemi et al. [48] explored VR-based training targeting executive functions in children with developmental coordination disorder. However, these findings should be interpreted cautiously due to the limited number of studies and methodological heterogeneity.

The predominance of positive findings in attention and executive functioning is consistent with the broader pediatric VR literature. A systematic review and meta-analysis by Corrigan et al. [50] found that immersive VR interventions in children with ADHD were associated with favorable effects on global cognition, attention, and memory, suggesting that virtual environments may be particularly suited to the rehabilitation of cognitive processes that require sustained engagement, rapid feedback, and repeated responses to dynamically changing stimuli. Similar findings have been reported outside strictly pediatric samples: a systematic review of fully immersive VR cognitive training across neurological and psychiatric disorders found that the most promising improvements occurred in executive functioning and attention, although the evidence was derived mainly from small pilot trials with methodological concerns [51]. These observations support the hypothesis that VR may facilitate executive rehabilitation by combining repetitive cognitive practice with immediate feedback, task adaptation, and environments that require active decision-making.

Nevertheless, the apparent effectiveness of VR for executive functioning should be interpreted cautiously. The studies included in the present review differed markedly in the individual executive processes targeted, and improvements in one component did not necessarily extend to other executive abilities or to everyday executive functioning. This distinction between improvement on specific neuropsychological measures and meaningful functional transfer is clinically important. Evidence from other rehabilitation populations similarly suggests that cognitive gains detected on standardized measures do not automatically imply equivalent improvements in activities of daily living [52]. Although meta-analytic evidence in adults with post-stroke cognitive impairment indicates that VR may improve both cognition and daily functioning [53], such functional generalization has not yet been demonstrated consistently in pediatric cognitive rehabilitation. Thus, future pediatric studies should move beyond demonstration of task-specific improvement and establish whether gains translate into classroom performance, autonomy, participation, and daily cognitive functioning.

The evidence for memory rehabilitation was comparatively limited in the present study. Memory was often incorporated as one component of multidomain executive or cognitive interventions rather than investigated as a primary rehabilitation target. This differs somewhat from the findings of Corrigan et al. [50], whose meta-analysis in pediatric ADHD suggested beneficial effects of immersive VR on memory as well as attention. The discrepancy may reflect differences in inclusion criteria and in how cognitive domains were classified. It also illustrates a broader problem in this literature: terms such as working memory, executive functioning, attention, and global cognition frequently overlap across intervention protocols and outcome batteries, making domain-specific conclusions difficult. More explicit theoretical models of the targeted cognitive process and predefined primary outcomes would improve comparability across future studies.

Language and cognitive-communication rehabilitation represented a smaller but particularly relevant component of the evidence base. The findings suggest that virtual environments can be incorporated successfully into receptive and expressive language treatment, but evidence for additional effectiveness over conventional therapy remains less established than feasibility. This interpretation is consistent with the broader digital-language literature. Zhou et al. [54], in a systematic review of digital interventions for children with developmental language disorder (DLD), identified 10 studies involving 961 children and found that phonological skills, general language functioning, grammar, and vocabulary were the principal targets. However, heterogeneity remained substantial, and evidence was insufficient to determine consistent effectiveness across different language domains, particularly expressive language [54]. Similarly, the more recent controlled VR-supported DLD study found good feasibility, safety, and usability but did not demonstrate a significant treatment-by-time advantage over conventional therapy across the language outcomes examined [49]. This does not diminish the potential clinical value of VR, but suggests that its role may be better conceptualized as a means of enriching and individualizing conventional speech-language rehabilitation rather than as a replacement for therapist-led treatment.

A similar pattern emerged for social cognition and socio-emotional functioning, particularly in children with ASD. The studies included in this review suggested possible benefits for emotion recognition, social attribution, and analogical reasoning. These findings agree with recent meta-analytic evidence showing favorable effects of immersive VR on cognitive, social, and emotional outcomes in children and adolescents with ASD [55]. Likewise, systematic reviews of VR-based social-skills interventions have generally reported promising outcomes [56]. However, contrasting evidence is also important. Roberts et al. [57] concluded that the quality of studies examining extended-reality social-skills interventions in autistic children was generally low and that there was insufficient evidence regarding generalization, maintenance, or meaningfulness of the observed effects to autistic individuals themselves. Therefore, improvements in laboratory-based emotion-recognition or social-cognition tasks should not automatically be interpreted as improvement in spontaneous real-world social communication.

Another notable finding concerns intervention characteristics and dose. Treatment protocols differed substantially, ranging from short programs comprising fewer than ten sessions to prolonged rehabilitation delivered for six months. The present evidence did not indicate a straightforward dose–response relationship. This is consistent with findings from other VR rehabilitation fields, where intervention frequency and duration appear important but do not show a simple linear relationship with effectiveness. For example, a meta-analysis comparing immersive and non-immersive VR rehabilitation in cognitive impairment suggested that interventions delivered for 5–8 weeks, for more than 30 min and at least three times per week, were associated with favorable outcomes, while longer exposure did not necessarily result in greater benefit [58]. These data arise from older adult populations and therefore cannot be directly extrapolated to children, but they reinforce the need to investigate treatment dose empirically rather than assuming that greater exposure is inherently more effective. In pediatric rehabilitation, developmental stage, fatigue, attention span, tolerability, and the complexity of the targeted function are likely to influence the optimal dose.

The heterogeneity of the control conditions also affects interpretation. Studies comparing VR with no intervention or a waitlist can establish whether training is associated with improvement but cannot determine whether the virtual component itself confers an advantage over an equivalent conventional intervention. This is particularly important because VR may increase novelty, motivation, therapist contact, or overall activity independently of its specific rehabilitation mechanisms. Findings from other pediatric rehabilitation fields illustrate this issue. Reviews of VR in pediatric rehabilitation, including cerebral palsy, have reported promising effects; however, they also indicate that VR is not consistently superior to active conventional treatments and may function most effectively as an adjunctive approach [59,60,61]. Accordingly, future cognitive and language trials should preferentially use dose-matched active comparators.

The comparison by level of immersion represents another important finding. The present review did not identify consistent evidence that immersive VR was more effective than semi-immersive or non-immersive systems. Screen- and smartphone-based interactive virtual environments were considered non-immersive VR when they involved interaction with computer-generated environments or virtual agents, consistent with the operational definition adopted in this review. Rather, different technologies appeared to be used for different rehabilitation goals. Immersive systems were more frequently used for attention or complex cognitive-neurological rehabilitation; semi-immersive systems often incorporated body motion and cognitive-motor tasks; and non-immersive systems were commonly used for language or social-cognition interventions. This finding supports the idea that technological sophistication should not be equated automatically with therapeutic superiority. Pediatric evidence on non-immersive VR indicates that such systems can be feasible, acceptable, and clinically useful while imposing fewer technical and sensory demands [61]. Evidence from adult populations is not entirely consistent with this interpretation. Ren et al. [58] reported that immersive VR produced larger effects than non-immersive VR for some cognitive and motor outcomes in individuals with mild cognitive impairment or dementia. However, these findings concern older adults and cannot establish that greater immersion is advantageous in pediatric neurorehabilitation. Children may respond differently to immersive environments because of developmental, sensory, attentional, and ergonomic factors. In addition, generational differences in familiarity with digital technologies may play a facilitating role [62]. Children and adolescents are generally exposed to interactive digital devices from an early age and may therefore approach VR interfaces with greater intuitiveness and adaptability than older adults, for whom limited previous exposure to digital technologies may represent an additional usability or learning barrier. This greater technological familiarity could contribute to engagement, adherence, and ease of interaction with virtual rehabilitation environments, independently of the level of immersion itself. At the same time, exposure duration should be carefully considered when designing pediatric VR protocols. Session length, frequency, and cumulative exposure should be adapted to the child’s age and developmental stage and aligned, where available, with age-appropriate recommendations for screen and immersive-technology use [63]. Particular attention should be paid to younger children, for whom shorter sessions, regular breaks, and close monitoring of visual discomfort, fatigue, dizziness, headache, and cybersickness may be especially important.

Moreover, non-immersive systems may offer practical advantages, including lower cost, easier therapist supervision, reduced equipment burden, and potentially greater suitability for younger children. Direct randomized comparisons of immersion levels within the same pediatric population and rehabilitation protocol are therefore required.

The relatively limited representation of AR further reinforces the predominance of VR in this research field. Although the AR study included in the review reported favorable executive and behavioral findings, there is currently insufficient pediatric cognitive-rehabilitation evidence to compare AR directly with VR. AR may offer a theoretically attractive compromise because virtual information can be embedded within real-world activities, potentially supporting ecological transfer without complete sensory isolation. However, this hypothesis remains largely untested in pediatric cognitive and linguistic rehabilitation.

The generally positive feasibility and tolerability findings are also clinically relevant. VR is often proposed as a way of increasing engagement with repetitive rehabilitation tasks, particularly in children. Broader pediatric reviews indicate that VR can be feasible across a range of medical contexts and may support engagement and experiential learning [64]. Nevertheless, feasibility should be distinguished from safety. Cybersickness—including nausea, dizziness, headache, visual discomfort, disorientation, and fatigue—remains a recognized adverse effect of VR exposure [65]. Pediatric systematic reviews also show that cybersickness screening and reporting remain inconsistent, even in immersive VR research involving children [66]. Thus, the absence of reported adverse events in individual rehabilitation studies may partly reflect inadequate assessment rather than complete absence of symptoms. Standardized pediatric measures of cybersickness, visual discomfort, fatigue, and acceptability should become routine outcomes in future trials.

### 4.1. Strengths and Limitations

This scoping review has several strengths. It adopted a broad developmental and rehabilitation perspective, integrating cognitive, linguistic, and cognitive-communication outcomes within a single framework rather than considering these domains separately. This is particularly relevant in childhood, where language, attention, memory, and executive functions are strongly interdependent. The search covered multiple biomedical, multidisciplinary, engineering, and intervention databases, including PubMed/MEDLINE, Web of Science, Cochrane Library, IEEE Xplore, Embase, PsycINFO and Scopus without publication-date restrictions. In addition, the review considered different levels of VR immersion and included AR as a related but secondary technology, thereby allowing comparison of technological approaches. Furthermore, intervention dose, outcome instruments, follow-up, and technological characteristics were analyzed. Several limitations should also be acknowledged. The review was restricted to English-language journal articles, potentially introducing language and publication bias and excluding relevant conference proceedings, which may be particularly important in rapidly developing technological fields. The included studies were highly heterogeneous in diagnoses, age ranges, intervention targets, VR configurations, treatment intensity, outcome measures, and comparators, precluding quantitative synthesis and limiting direct cross-study comparisons. Sample sizes were generally small, and several studies used uncontrolled, feasibility, pilot, or single-case designs. Blinding was frequently difficult or incompletely reported, and inactive control groups were common. Follow-up assessments were rare, limiting conclusions regarding durability. Furthermore, some interventions combined VR with conventional rehabilitation, exercise, pharmacological management, or therapist-delivered treatment, making it difficult to isolate the independent contribution of VR. Finally, because this was a scoping review, the findings should be interpreted as a mapping of the available evidence and not as proof of comparative treatment efficacy.

### 4.2. Future Perspectives

Future research should prioritize well-powered, multicenter randomized controlled trials with clearly prespecified cognitive or linguistic targets and dose-matched active comparators. Greater standardization of intervention reporting is needed, including hardware configuration, field of view, tracking system, interaction modality, level of immersion, therapist involvement, session frequency and duration, adaptive algorithms, and home versus clinical delivery. Such information is essential for reproducibility and for identifying which technological components actually contribute to therapeutic benefit.

A second priority is the adoption of core outcome sets for pediatric VR cognitive rehabilitation. Studies should combine standardized performance-based measures with ecologically meaningful outcomes reflecting school performance, communication, autonomy, participation, and caregiver- or teacher-observed functioning. Longitudinal follow-up should be incorporated routinely to distinguish transient training effects from durable rehabilitation gains. Future trials should also directly compare immersive, semi-immersive, and non-immersive systems using equivalent therapeutic content. This would allow investigators to determine whether immersion itself contributes to rehabilitation or whether outcomes are principally driven by repetition, feedback, adaptivity, and engagement. Cost-effectiveness and implementation studies are also needed because the most immersive platform may not be the most scalable solution for schools, outpatient clinics, or home rehabilitation. Future VR language programs should investigate lexical access, phonology, morphosyntax, narrative abilities, discourse, and pragmatic communication while integrating measures of attention and executive functioning to reflect the cognitive-linguistic nature of language rehabilitation. Finally, safety and developmental appropriateness should be embedded within intervention design rather than treated as secondary considerations. Trials should systematically collect cybersickness, headache, visual discomfort, fatigue, postural symptoms, dropout, usability, adherence, and child-reported acceptability. Age-specific hardware fit, developmental capacity to understand virtual environments, sensory sensitivities, and accessibility for neurodevelopmentally diverse children should also be considered. Participatory design involving children, caregivers, therapists, and educators may help ensure that future systems target clinically meaningful goals rather than technological novelty alone.

## 5. Conclusions

VR represents a promising and versatile approach to cognitive and linguistic rehabilitation in children and adolescents. The available literature suggests potential benefits across attention, executive functions, selected memory processes, language, and social cognition, with the most consistent evidence currently concerning attention and executive functioning. Beneficial findings have been reported across immersive, semi-immersive, and non-immersive systems, and the current evidence does not establish that greater immersion produces superior rehabilitation outcomes. Language and social-cognitive applications are promising but remain less extensively investigated, while AR evidence is still preliminary.

Overall, VR should currently be viewed as an adjunctive and adaptable rehabilitation tool rather than a replacement for established therapist-delivered interventions. The field now requires a transition from small exploratory studies toward adequately powered trials that use standardized outcomes, active comparators, long-term follow-up, rigorous safety monitoring, and clear technological reporting. Establishing whether improvements generalize from trained virtual tasks to everyday cognitive, communicative, academic, and social functioning will be crucial for determining the true clinical value of VR in pediatric rehabilitation.

## Figures and Tables

**Figure 1:**
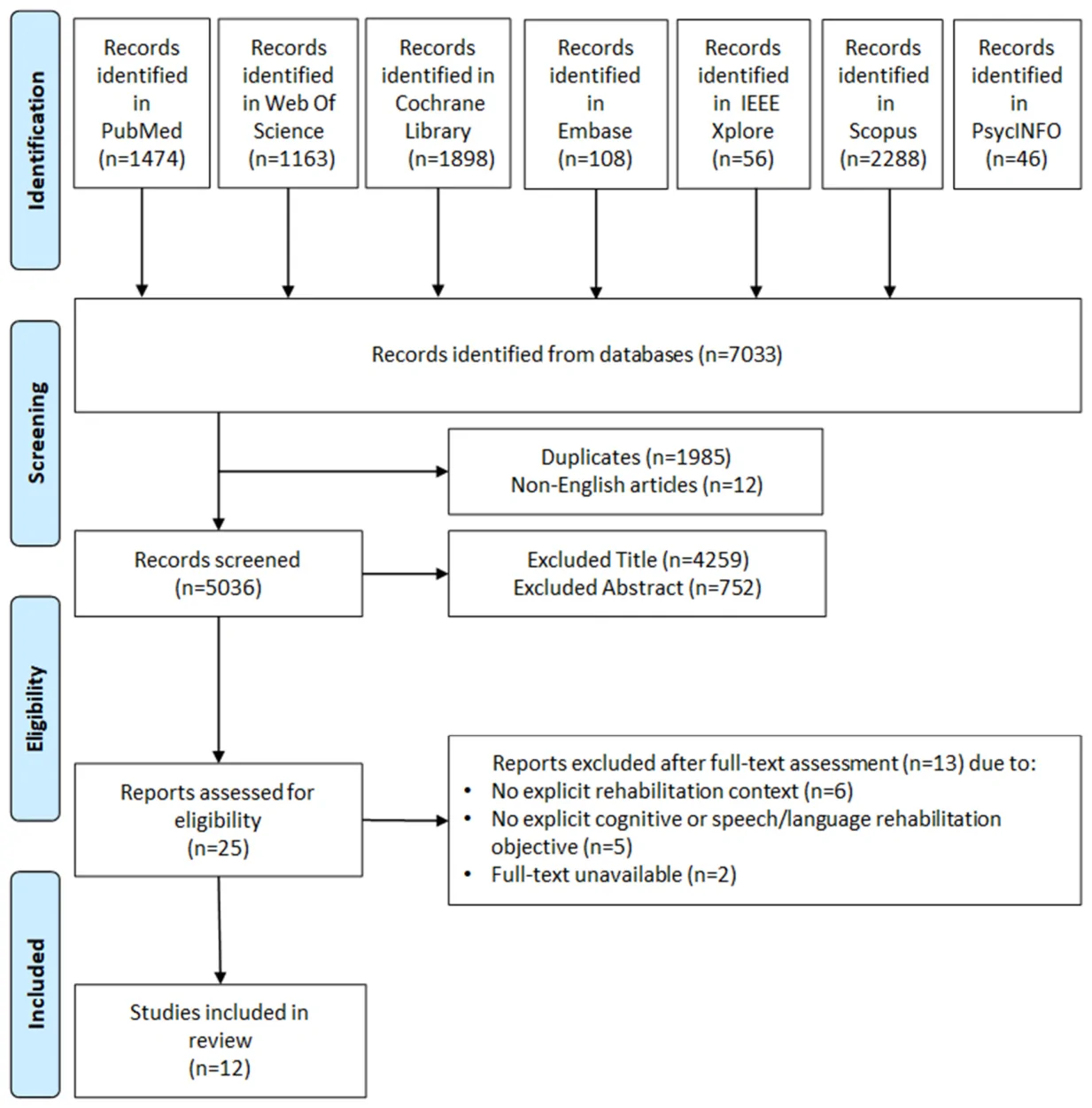
PRISMA-ScR flow diagram of study identification, screening, eligibility, and inclusion.

**Table 1:** Characteristics of the included studies.

Reference	Country	Population	Study Design	VR/AR Technology	Intervention	Domains	Results	Limitations
Cho et al., 2002 [38]	South Korea	Children/adolescents; exact age range NR; N = 30	Randomized controlled trial	Head-mounted display-based cognitive-training system with position tracking; immersive VR	8 sessions over 2 weeks; session duration NR	Sustained attention, attentional control	The immersive VR group showed greater improvement in sustained attention than the non-VR computerized training and no-intervention groups.	Small sample; age insufficiently characterized; brief intervention; no follow-up
Didehbani et al., 2016 [39]	USA	7–16 years; N = 30, ASD	Uncontrolled pre–post study	Desktop monitor-based avatar-mediated virtual social environment; non-immersive VR	10 sessions, 60 min each, over 5 weeks	Emotion recognition, social attribution, analogical reasoning, executive functioning	VR-based social-cognition training was associated with improvements in emotion recognition, social attribution, and analogical reasoning.	No control group; small selected sample; potential practice and expectancy effects
Cooper et al., 2018 [40]	Australia	17 years; N = 1, female with chronic memory impairment	Single-case ABA design	Digitally delivered virtual peer-supported intervention; non-immersive VR	Daily use for 3 weeks; daily duration NR	Autobiographical memory, prospective memory, event recall, everyday functioning	The intervention was associated with improved event recall and better self-reported everyday memory functioning.	Single case; no active comparator; limited generalizability
De Luca et al., 2020 [41]	Italy	15 years; N = 1, male with severe TBI	Case report	Projection-based computer-assisted rehabilitation environment with motion capture and instrumented platform; immersive VR	Conventional rehabilitation followed by VR-assisted phase; exact number and duration NR	Attention, visuospatial abilities, executive functions, behavioral functioning	Cognitive and behavioral outcomes improved following the immersive VR rehabilitation phase, particularly in attention, visuospatial abilities, and executive functioning.	Single case; sequential design; no randomization; possible order and carryover effects
Shen et al., 2022 [42]	USA	7–17 years; N = 26, TBI	Parallel-group randomized feasibility/safety trial	Interactive head-/body-tracked cognitive rehabilitation environment; immersive VR	At least one VR training session; approximately 20–30 min/session; participants could complete additional sessions	Executive functions, attention, working memory, processing speed	The VR intervention was feasible and well tolerated and showed preliminary evidence of improvement in cognitive functions, including executive abilities and attention.	Small feasibility sample; not powered for definitive efficacy; limited follow-up
Cappadona et al., 2023 [43]	Italy	3–7 years; N = 32, developmental language disorder	Pilot randomized controlled trial	Touchscreen-based interactive virtual rehabilitation system; non-immersive VR	48 sessions; 2/week, 60 min/session, over 6 months	Receptive and expressive language	Both groups improved, but children receiving VR-based speech-language therapy showed greater gains in several receptive and expressive language abilities, particularly comprehension, naming, morphosyntax, sentence construction, and spontaneous language.	Small single-center pilot; multiple outcomes; no long-term post-treatment follow-up
Di Giusto et al., 2023 [44]	Italy	7–11 years; N = 24, specific learning disorders	Uncontrolled pilot study with 6-month follow-up	Projection- and motion-tracking-based interactive rehabilitation environment; semi-immersive VR	6-week intervention; frequency/session duration NR	Visual attention, inhibition, flexibility, planning	Significant improvements were observed in visual attention, inhibition, cognitive flexibility, and planning, with several benefits maintained at six-month follow-up.	No control group; small sample; possible maturation and practice effects
Zhao et al., 2024 [45]	China	6–12 years; N = 90, ADHD	Randomized controlled trial	Motion-based cognitive-physical system overlaying virtual elements onto real-world activity; AR	12 sessions, 30 min/session, 3/week, over 4 weeks	Executive functioning, metacognition, behavioral regulation	The AR-based intervention produced improvements in metacognition, global executive functioning, and ADHD-related symptoms compared with the control condition.	Combined cognitive and physical components; mainly parent-rated outcomes; no long-term follow-up
Lyu et al., 2024 [46]	China	Adolescents with mild-to-moderate depressive episodes; N = 137 randomized: VR n = 47, waitlist n = 46; healthy comparison n = 44	Single-blind pilot RCT	Computer-generated cognitive-training environment; non-immersive VR	20 sessions over 7 weeks; 30 min/session	Planning, attention, simultaneous processing, successive processing, working memory	VR-based cognitive training improved planning, attention, simultaneous and successive processing, and working memory, together with a reduction in depressive symptoms.	Waitlist rather than active comparator; single center; no extended follow-up; immersion level unclear
Abdullah et al., 2024 [47]	Malaysia	Children with Autism Spectrum Disorder; N = 21; 7–14 years	Pre–post intervention study	Mobile/tablet-based AR	AR-based cognitive rehabilitation program using interactive 3D objects and visual/auditory feedback; identification of 15 objects	Attention, concentration, object identification/cognitive learning	Improvements were reported in cognitive-learning performance following AR-based training.	No control group; small sample; pre–post design; limited outcome measures
Hashemi et al., 2025 [48]	Iran	Children with Developmental Coordination Disorder; N = 40 boys; 7–9 years; VR group n = 20, control group n = 20	Semi-experimental pre–post intervention study	Xbox Kinect 360-based virtual reality training; semi-immersive VR	VR training for 8 weeks; 2 sessions/week; 30 min/session	Executive functions, cognitive dominance, problem solving/planning, behavioral-emotional organization	VR training significantly improved executive functions and cognitive dominance compared with the control condition.	Small sample; only boys included; limited follow-up
De Domenico et al., 2026 [49]	Italy	3–7 years; N = 56; VR n = 28, control n = 28; developmental language disorder	Randomized controlled pilot study	Touchscreen-based interactive virtual rehabilitation system; non-immersive VR	48 sessions; 2/week, 60 min/session, over 6 months	Receptive and expressive language	Both VR-supported and conventional speech-language therapy produced improvements in language abilities. No clear between-group superiority emerged, while VR treatment showed excellent retention and no reported adverse events.	Single center; no superiority over conventional therapy on between-group analyses; limited longer-term follow-up

Legend: ADHD = attention-deficit/hyperactivity disorder; AR = augmented reality; ASD = autism spectrum disorder; NR = not reported; RCT = randomized controlled trial; TBI = traumatic brain injury; VR = virtual reality.

**Table 2:** Distribution of VR/AR technologies across pediatric populations and rehabilitation outcomes.

Participant Group	VR/AR Type	Included Studies	Main Rehabilitation Outcomes
Autism spectrum disorder	Non-immersive VR	Didehbani et al., 2016 [39]	Social cognition, emotion recognition, social attribution, executive functioning
Autism spectrum disorder	AR	Abdullah et al., 2024 [47]	Attention, concentration, object identification/cognitive learning
Traumatic brain injury	Immersive VR	De Luca et al., 2020 [41]; Shen et al., 2022 [42]	Attention, visuospatial abilities, executive functions, working memory, processing speed
Developmental coordination disorder	Semi-immersive VR	Hashemi et al., 2025 [48]	Executive functions and cognitive skills
Speech/language disorders	Non-immersive VR	Cappadona et al., 2023 [43]; De Domenico et al., 2026 [49]	Receptive and expressive language
Attention/cognitive difficulties	Immersive VR	Cho et al., 2002 [38]	Attention, attentional control
ADHD	Non-immersive VR	Zhao et al., 2024 [45]	Executive functioning, metacognition, behavioral regulation
Specific learning disorders	Semi-immersive VR	Di Giusto et al., 2023 [44]	Visual attention, inhibition, flexibility, planning
Memory impairment	Non-immersive VR	Cooper et al., 2018 [40]	Autobiographical and prospective memory, event recall, everyday memory functioning
Depressive episodes	Non-immersive VR	Lyu et al., 2024 [46]	Planning, attention, simultaneous processing, successive processing, working memory

Legend: AR = augmented reality; VR = virtual reality.

## Data Availability

No new data were created or analyzed in this study. Data sharing is not applicable to this article.

## Supplementary Materials

The following supporting information can be downloaded at https://www.mdpi.com/article/10.3390/healthcare14193299/s1. Table S1. Database-specific search strategies; Table S2. Summary of the JBI critical appraisal results for randomized controlled trials; Table S3. Summary of the JBI critical appraisal results for quasi-experimental and uncontrolled intervention studies; Table S4. Summary of the JBI critical appraisal results for case reports and single-case studies; Table S5. PRISMA-ScR Checklist for reporting scoping reviews.
