# Supplementary material for: Virtual and Augmented Reality for Cognitive and Language Rehabilitation in Children and Adolescents: A Scoping Review
Source: Healthcare (Basel). 2026 Oct 3;14(19):3299. doi: 10.3390/healthcare14193299 (PMC13665212; doi:10.3390/healthcare14193299)
Supplement: Supplementary file 1 [file healthcare-14-03299-s001.zip › healthcare-4565537-supplementary.pdf]

Virtual and Augmented Reality for Cognitive and Language Rehabilitation in Children and Adolescents: A Scoping Review

Supplementary Materials

Table S1. Search Strings

Database	Search string
**PubMed**	(Child[Mesh] OR Adolescent[Mesh] OR child OR children OR adolescent OR adolescents OR pediatric OR paediatric OR youth) AND ("cognitive rehabilitation" OR "cognitive remediation" OR "cognitive training" OR "language rehabilitation" OR "speech therapy" OR "attention training" OR "executive function training" OR "memory training" OR "working memory training" OR ((cognition OR attention OR "executive function" OR memory OR language OR speech OR communication) AND (rehabilitation OR remediation OR training OR stimulation OR therapy OR intervention))) AND ("Virtual Reality"[Mesh] OR "Augmented Reality"[Mesh] OR "virtual reality" OR "virtual environment" OR "immersive virtual reality" OR "semi-immersive virtual reality" OR "non-immersive virtual reality" OR "head-mounted display" OR "augmented reality" OR "mixed reality")
**Web of Science**	(child OR children OR adolescent OR adolescents OR pediatric OR paediatric OR youth) AND ("cognitive rehabilitation" OR "cognitive remediation" OR "cognitive training" OR "language rehabilitation" OR "speech therapy" OR "attention training" OR "executive function training" OR "memory training" OR "working memory training" OR ((cognition OR attention OR "executive function" OR memory OR language OR speech OR communication) AND (rehabilitation OR remediation OR training OR stimulation OR therapy OR intervention))) AND ("virtual reality" OR "virtual environment" OR "immersive virtual reality" OR "semi-immersive virtual reality" OR "non-immersive virtual reality" OR "head-mounted display" OR "augmented reality" OR "mixed reality")
**Cochrane Library**	(child OR children OR adolescent OR adolescents OR pediatric OR paediatric OR youth) AND ("cognitive rehabilitation" OR "cognitive remediation" OR "cognitive training" OR "language rehabilitation" OR "speech therapy" OR "attention training" OR "executive function training" OR "memory training" OR "working memory training" OR ((cognition OR attention OR "executive function" OR memory OR language OR speech OR communication) AND (rehabilitation OR remediation OR training OR stimulation OR therapy OR intervention))) AND ("virtual reality" OR "virtual environment" OR "immersive virtual reality" OR "semi-immersive virtual reality" OR "non-immersive virtual reality" OR "head-mounted display" OR "augmented reality" OR "mixed reality")
**IEEE Xplore**	("All Metadata":child OR "All Metadata":children OR "All Metadata":adolescent OR "All Metadata":adolescents OR "All Metadata":pediatric OR "All Metadata":paediatric OR "All Metadata":youth) AND ("All Metadata":"cognitive rehabilitation" OR "All Metadata":"cognitive remediation" OR "All Metadata":"cognitive training" OR "All Metadata":"language rehabilitation" OR "All Metadata":"speech therapy" OR "All Metadata":"attention training" OR "All Metadata":"executive function training" OR "All Metadata":"memory training" OR "All Metadata":"working memory training") AND ("All Metadata":"virtual reality" OR "All Metadata":"virtual environment" OR "All Metadata":"immersive virtual reality" OR "All Metadata":"augmented reality" OR "All Metadata":"mixed reality")

**Embase**	('child'/exp OR 'adolescent'/exp OR child:ti,ab,kw OR children:ti,ab,kw OR adolescent:ti,ab,kw OR adolescents:ti,ab,kw OR pediatric:ti,ab,kw OR paediatric:ti,ab,kw OR youth:ti,ab,kw) AND ('cognitive rehabilitation'/exp OR 'cognitive training'/exp OR 'cognitive rehabilitation':ti,ab,kw OR 'cognitive remediation':ti,ab,kw OR 'cognitive training':ti,ab,kw OR 'language rehabilitation':ti,ab,kw OR 'speech therapy':ti,ab,kw OR 'attention training':ti,ab,kw OR 'executive function training':ti,ab,kw OR 'memory training':ti,ab,kw OR 'working memory training':ti,ab,kw) AND ('virtual reality'/exp OR 'augmented reality'/exp OR 'virtual reality':ti,ab,kw OR 'virtual environment':ti,ab,kw OR 'immersive virtual reality':ti,ab,kw OR 'semi-immersive virtual reality':ti,ab,kw OR 'non-immersive virtual reality':ti,ab,kw OR 'head-mounted display':ti,ab,kw OR 'augmented reality':ti,ab,kw OR 'mixed reality':ti,ab,kw)
**PsycINFO**	(Any Field: child OR Any Field: children OR Any Field: adolescent OR Any Field: adolescents OR Any Field: pediatric OR Any Field: paediatric OR Any Field: youth) AND (Any Field: "cognitive rehabilitation" OR Any Field: "cognitive remediation" OR Any Field: "cognitive training" OR Any Field: "language rehabilitation" OR Any Field: "speech therapy" OR Any Field: "attention training" OR Any Field: "executive function training" OR Any Field: "memory training" OR Any Field: "working memory training" OR ((Any Field: cognition OR Any Field: attention OR Any Field: "executive function" OR Any Field: memory OR Any Field: language OR Any Field: speech OR Any Field: communication) AND (Any Field: rehabilitation OR Any Field: remediation OR Any Field: training OR Any Field: stimulation OR Any Field: therapy OR Any Field: intervention))) AND (Any Field: "virtual reality" OR Any Field: "virtual environment" OR Any Field: "immersive virtual reality" OR Any Field: "semi-immersive virtual reality" OR Any Field: "non-immersive virtual reality" OR Any Field: "head-mounted display" OR Any Field: "augmented reality" OR Any Field: "mixed reality")
**Scopus**	(child OR children OR adolescent OR adolescents OR pediatric OR paediatric OR youth) AND ("cognitive rehabilitation" OR "cognitive remediation" OR "cognitive training" OR "language rehabilitation" OR "speech therapy" OR "attention training" OR "executive function training" OR "memory training" OR "working memory training" OR ((cognition OR attention OR "executive function" OR memory OR language OR speech OR communication) AND (rehabilitation OR remediation OR training OR stimulation OR therapy OR intervention))) AND ("virtual reality" OR "virtual environment" OR "immersive virtual reality" OR "semi-immersive virtual reality" OR "non-immersive virtual reality" OR "head-mounted display" OR "augmented reality" OR "mixed reality")"

Table S2. Summary of the JBI critical appraisal results for randomized controlled trials

Item JBI Checklist	JBI Quality Assessment Question	Study References					
		Cho et al. (2002)	Shen et al. (2022)	Cappadona et al. (2023)	Zhao et al. (2024)	Lyu et al. (2024)	De Domenico et al. (2026)
1	Was true randomization used for assignment of participants to treatment groups?	Unclear	Yes	Yes	Yes	Unclear	Yes
2	Was allocation to treatment groups concealed?	Unclear	Unclear	Unclear	Unclear	Unclear	Unclear
3	Were treatment groups similar at baseline?	Unclear	Yes	Yes	Yes	Yes	Yes
4	Were participants blind to treatment assignment?	No	No	No	No	No	No
5	Were those delivering treatment blind to treatment assignment?	No	No	No	No	No	No
6	Were outcome assessors blind to treatment assignment?	Unclear	Unclear	Yes	No	Yes	Yes
7	Were treatment groups treated identically other than the intervention of interest?	Unclear	Yes	Yes	No	No	Yes
8	Was follow-up complete and, if not, were differences between groups in terms of follow-up adequately described and analyzed?	Unclear	Yes	Yes	Yes	Yes	Yes
9	Were participants analyzed in the groups to which they were randomized?	Unclear	Yes	Yes	No	Unclear	Yes
10	Were outcomes measured in the same way for treatment groups?	Yes	Yes	Yes	Yes	Yes	Yes
11	Were outcomes measured in a reliable way?	Yes	Yes	Yes	Yes	Yes	Yes
12	Was appropriate statistical analysis used?	Unclear	Yes	Yes	Yes	Yes	Yes
13	Was the trial design appropriate, and were any deviations from the standard RCT design accounted for in the conduct and analysis of the trial?	Yes	Yes	Yes	Yes	Yes	Yes

Legend: Yes = criterion satisfied; No = criterion not satisfied; Unclear = insufficient reporting; N/A = not applicable.

Table S3. Summary of the JBI critical appraisal results for quasi-experimental and uncontrolled intervention studies

Item JBI Checklist	JBI Quality Assessment Question	Study References			
		Didehbani et al. (2016)	Di Giusto et al. (2023)	Abdullah et al. (2024)	Hashemi et al. (2025)
1	Is it clear in the study what is the "cause" and what is the "effect" (i.e., there is no confusion about which variable comes first)?	Yes	Yes	Yes	Yes
2	Were the participants included in any comparisons similar?	N/A	N/A	Yes	Yes
3	Were the participants included in any comparisons receiving similar treatment/care, other than the exposure or intervention of interest?	N/A	N/A	Yes	Yes
4	Was there a control group?	No	No	Yes	Yes
5	Were there multiple measurements of the outcome both pre and post the intervention/exposure?	No	No	Yes	Yes
6	Was follow-up complete and, if not, were differences between groups in terms of follow-up adequately described and analyzed?	Yes	Yes	Yes	Yes
7	Were the outcomes of participants included in any comparisons measured in the same way?	Yes	Yes	Yes	Yes
8	Were outcomes measured in a reliable way?	Yes	Yes	Yes	Yes
9	Was appropriate statistical analysis used?	Yes	Yes	Yes	Yes

Legend: Yes = criterion satisfied; No = criterion not satisfied; Unclear = insufficient reporting; N/A = not applicable.

Table S4. Summary of the JBI critical appraisal results for case reports and single-case studies

Item JBI Checklist	JBI Quality Assessment Question	Study References	
		**Cooper et al. (2018)**	**De Luca et al. (2020)**
**1**	Were the patient's demographic characteristics clearly described?	**Yes**	**Yes**
**2**	Was the patient's history clearly described and presented as a timeline?	**Yes**	**Yes**
**3**	Was the current clinical condition of the patient on presentation clearly described?	**Yes**	**Yes**
**4**	Were diagnostic tests or assessment methods and the results clearly described?	**Yes**	**Yes**
**5**	Was the intervention(s) or treatment procedure(s) clearly described?	**Yes**	**Yes**
**6**	Was the post-intervention clinical condition clearly described?	**Yes**	**Yes**
**7**	Were adverse events (harms) or unanticipated events identified and described?	**Unclear**	**Unclear**
**8**	Does the case report provide takeaway lessons?	**Yes**	**Yes**

Legend: Yes = criterion satisfied; No = criterion not satisfied; Unclear = insufficient reporting; N/A = not applicable.

Table S5. Preferred Reporting Items for Systematic reviews and Meta-Analyses extension for Scoping Reviews (PRISMA-ScR) Checklist

SECTION	ITEM	PRISMA-ScR CHECKLIST ITEM	REPORTED ON PAGE #
**TITLE**			
Title	1	Identify the report as a scoping review.	Page 1
**ABSTRACT**			
Structured summary	2	Provide a structured summary that includes (as applicable): background, objectives, eligibility criteria, sources of evidence, charting methods, results, and conclusions that relate to the review questions and objectives.	Page 1
**INTRODUCTION**			
Rationale	3	Describe the rationale for the review in the context of what is already known. Explain why the review questions/objectives lend themselves to a scoping review approach.	Pages 2-4
Objectives	4	Provide an explicit statement of the questions and objectives being addressed with reference to their key elements (e.g., population or participants, concepts, and context) or other relevant key elements used to conceptualize the review questions and/or objectives.	Page 4
**METHODS**			
Protocol and registration	5	Indicate whether a review protocol exists; state if and where it can be accessed (e.g., a Web address); and if available, provide registration information, including the registration number.	Page 4
Eligibility criteria	6	Specify characteristics of the sources of evidence used as eligibility criteria (e.g., years considered, language, and publication status), and provide a rationale.	Pages 4-6
Information sources*	7	Describe all information sources in the search (e.g., databases with dates of coverage and contact with authors to identify additional sources), as well as the date the most recent search was executed.	Pages 4-6
Search	8	Present the full electronic search strategy for at least 1 database, including any limits used, such that it could be repeated.	Pages 4-6
Selection of sources of evidence†	9	State the process for selecting sources of evidence (i.e., screening and eligibility) included in the scoping review.	Pages 4-6
Data charting process‡	10	Describe the methods of charting data from the included sources of evidence (e.g., calibrated forms or forms that have been tested by the team before their use, and whether data charting was done independently or in duplicate) and any processes for obtaining and confirming data from investigators.	Pages 4-6
Data items	11	List and define all variables for which data were sought and any assumptions and simplifications made.	Pages 4-6
Critical appraisal of individual sources of evidence§	12	If done, provide a rationale for conducting a critical appraisal of included sources of evidence; describe the methods used and how this information was used in any data synthesis (if appropriate).	Pages 4-6
Synthesis of results	13	Describe the methods of handling and summarizing the data that were charted.	Pages 7-15
**RESULTS**			
Selection of sources of evidence	14	Give numbers of sources of evidence screened, assessed for eligibility, and included in the review, with reasons for exclusions at each stage, ideally using a flow diagram.	Pages 7-15
Characteristics of sources of evidence	15	For each source of evidence, present characteristics for which data were charted and provide the citations.	Pages 7-15

SECTION	ITEM	PRISMA-ScR CHECKLIST ITEM	REPORTED ON PAGE #
Critical appraisal within sources of evidence	16	If done, present data on critical appraisal of included sources of evidence (see item 12).	Pages 7-15
Results of individual sources of evidence	17	For each included source of evidence, present the relevant data that were charted that relate to the review questions and objectives.	Pages 7-15
Synthesis of results	18	Summarize and/or present the charting results as they relate to the review questions and objectives.	Pages 7-15
**DISCUSSION**			
Summary of evidence	19	Summarize the main results (including an overview of concepts, themes, and types of evidence available), link to the review questions and objectives, and consider the relevance to key groups.	Pages 15-20
Limitations	20	Discuss the limitations of the scoping review process.	Pages 15-20
Conclusions	21	Provide a general interpretation of the results with respect to the review questions and objectives, as well as potential implications and/or next steps.	Pages 20
**FUNDING**			
Funding	22	Describe sources of funding for the included sources of evidence, as well as sources of funding for the scoping review. Describe the role of the funders of the scoping review.	Page 21
